# The Impact of Sex Differences on Odor Identification and Facial Affect Recognition in Patients with Schizophrenia Spectrum Disorders

**DOI:** 10.3389/fpsyt.2018.00009

**Published:** 2018-01-31

**Authors:** Nilufar Mossaheb, Rainer M. Kaufmann, Monika Schlögelhofer, Thushara Aninilkumparambil, Claudia Himmelbauer, Anna Gold, Sonja Zehetmayer, Holger Hoffmann, Harald C. Traue, Harald Aschauer

**Affiliations:** ^1^Clinical Division of Social Psychiatry, Department of Psychiatry and Psychotherapy, Medical University Vienna, Vienna, Austria; ^2^Kepler University Clinic, Neuromed Campus, Linz, Austria; ^3^Department of Child and Adolescent Psychiatry, Medical University Vienna, Vienna, Austria; ^4^Penn Hospital – Black Country Partnership NHS Foundation Trust, Wolverhampton, United Kingdom; ^5^Department of Internal Medicine, Hospital Amstetten, Lower Austria, Austria; ^6^Hospital Oberpullendorf, Oberpullendorf, Burgenland, Austria; ^7^Section of Medical Statistics, Medical University Vienna, Vienna, Austria; ^8^Department of Psychosomatic Medicine and Psychotherapy, Section for Medical Psychology, University Ulm, Ulm, Germany; ^9^Biopsychosocial Corporation BioPsyC, Vienna, Austria

**Keywords:** schizophrenia, emotion recognition, smell identification, social cognition, sex

## Abstract

**Background:**

Social interactive functions such as facial emotion recognition and smell identification have been shown to differ between women and men. However, little is known about how these differences are mirrored in patients with schizophrenia and how these abilities interact with each other and with other clinical variables in patients vs. healthy controls.

**Methods:**

Standardized instruments were used to assess facial emotion recognition [Facially Expressed Emotion Labelling (FEEL)] and smell identification [University of Pennsylvania Smell Identification Test (UPSIT)] in 51 patients with schizophrenia spectrum disorders and 79 healthy controls; furthermore, working memory functions and clinical variables were assessed.

**Results:**

In both the univariate and the multivariate results, illness showed a significant influence on UPSIT and FEEL. The inclusion of age and working memory in the MANOVA resulted in a differential effect with sex and working memory as remaining significant factors. Duration of illness was correlated with both emotion recognition and smell identification in men only, whereas immediate general psychopathology and negative symptoms were associated with emotion recognition only in women.

**Conclusion:**

Being affected by schizophrenia spectrum disorder impacts one’s ability to correctly recognize facial affects and identify odors. Converging evidence suggests a link between the investigated basic and social cognitive abilities in patients with schizophrenia spectrum disorders with a strong contribution of working memory and differential effects of modulators in women vs. men.

## Introduction

Humans are social beings who have developed the ability to interpret complex and intertwined social contexts using a large array of cues. In fact, the recognition of non-verbal facial expressions of emotion is essential for social interaction and is an important component of social cognition. Similarly, detection of olfactory signals constitutes an essential part of social behavior in many animals. From an evolutionary perspective olfaction is in fact thought to be the oldest sense (1). Schizophrenia has been shown to be associated with impairments in basic and social cognition, which share some neural circuitry. Deficits in emotion recognition (ER) and smell identification [SI; smell identification deficits (SIDs)] have often been detected in chronic (2), first episode (3), and high-risk patients (4) as well as in unaffected relatives (5, 6) [for SID (7)]. Gender dichotomous differences in these domains have been shown in healthy subjects mostly in women showing higher emotional and olfactory discriminative abilities (8, 9). Similarly, in many, but not all studies, women with schizophrenia have been shown to outperform men with respect to both ER processes and olfactory identification (10, 11). However, some inconsistencies have been reported, which might, among other reasons, be explained by the heterogeneity of instruments used for the assessment of ER and SID in the different studies (2, 12).

Both, SI and ER are brain functions involved in social interaction and are connected to neurocognitive functions (13, 14); both are mediated through overlapping and interconnected brain regions including orbitofrontal and temporal regions. The intertwinement of emotional and olfactory processes has been shown in studies examining the hedonic appraisal of different odors (12), showing impairment in the appraisal of pleasant odors in patients with schizophrenia (15); a deficit that seems more prominent in men (16). In a small study investigating the relationship of ER and SI in 19 patients with schizophrenia and 14 controls, patients showed that poorer performances on facial affect recognition but not on odor detection thresholds or identification tasks (17). Neither sex nor severity of symptoms was modulating factors (17). However, there was a relationship between facial ER and unirhinal right nostril performance that was larger in patients than in control subjects and was apparent specifically for sad faces. To our knowledge, no other study has investigated the relationship between these social cognitive domains and possible gender dimorphism, while taking into account potentially influencing factors such as age and cognitive functions. Both ER and SID have been shown to be impacted by age (9, 18, 19) and to be associated with at least some aspects of neurocognitive functioning (14, 20). Thus, this analysis aimed to shed further light on the relationship between ER and SID with respect to possible gender differences in patients with schizophrenia spectrum disorders compared to healthy controls.

## Materials and Methods

### Sample

Inpatients and outpatients of the Department of Psychiatry and Psychotherapy of the Medical University of Vienna, Austria, with a DSM-IV-TR diagnosis of schizophrenia, schizophreniform, or schizoaffective disorder aged between 18 and 60 years were recruited. Exclusion criteria were organic mental disorder, mental retardation, pregnancy or lactation at time of assessment, clinically significant, non-corrected impairment of eyesight or of the olfactory system, acute medical condition (e.g., common cold), clinically significant dermatological diseases, atopic syndrome, use of anti-inflammatory, anti-allergic drugs or non-prescribed drugs, and alcohol at the time of assessments.

The sample was compared to healthy controls, recruited through concentric circle recruitment. Being healthy was defined as the absence of current or past psychiatric and organic disorders and of a family history of schizophrenia spectrum disorders. In addition to these, the same exclusion criteria as for patients were applied.

The data presented here are derived from a larger study, which was approved by the local ethics committee. The study was carried out in accordance with their recommendations. Written informed consent was obtained from all subjects before inclusion after thorough explanation of the study procedure in accordance with the Declaration of Helsinki. Furthermore, an unpublished master thesis derived from these data (21).

### Assessments

Data regarding sociodemographic and psychiatric history were collected in all participants.

Psychopathology was assessed by a trained rater using the Positive and Negative Syndrome Scale (PANSS) (22), a widely used semistructured interview assessing positive and negative symptoms of schizophrenia and global psychopathology. All raters were trained adequately, and interrater consistencies were assessed within team.

Clinical interview, medical records where available, and the Mini-International Neuropsychiatric Interview (MINI German Version 5.0.0) (23) were used for the assessment of diagnosis, which was done by consensus between two psychiatrists. The MINI is a short, structured interview, applicable by non-specialized rater and used to determine the occurrence of 17 psychiatric disorders.

Facial ER was assessed with the Facially Expressed Emotion Labelling (FEEL) test (24), a computerized assessment using color photographs of different people’s faces from the so-called JACFEE (Japanese and Caucasian Facial Expression of Emotion) series developed by Matsumoto and Ekman in 1988 (25) [cited in Ref. (24)]. The six displayed basic emotions are as follows: anger, sadness, fear, disgust, happiness, and surprise. The FEEL test starts with an emotionally neutral face. The same face then reappears for 300 ms displaying one of the above-mentioned emotions. The task consists of correctly identifying the shown emotion. A total of 42 pictures are presented. The maximum achievable score is 42. The FEEL test shows a high reliability (Cronbach’s coefficient of *r* = 0.77) compared to other published ER tests (24). Healthy women have been shown to outperform men in subtle expressions of emotions in the FEEL test but not in faces with high emotional expressiveness (26).

Smell identification deficits were assessed using the standardized University of Pennsylvania Smell Identification Test (UPSIT) German version (27, 28). A total of 40 odorants is equally divided onto four scratch and sniff booklets. The odorant is embedded in the microcapsules of 10–50 µg in diameter located on scent strips. The test is forced choice and multiple choice. A broad range of single and multiple odorants that can be divided into different classes are used. The UPSIT has been shown to be highly reliable and internally consistent (29). Normal ranges for the UPSIT are 35–40 for men and 34–40 for women; scores below these ranges are signs of olfactory identification impairments. Healthy women have been shown to outperform men in the UPSIT (29).

Intellectual functioning, specifically an estimate of premorbid intelligence, was assessed with the “Mehrfachwahl-Wortschatz-Intelligenztest” [MWT-B; Multiple-Choice Word Test-B (30), a quick and easy-to-administer multiple-choice word test with 37 items (31)]. The MWT-B was administered to exclude differences that might be explained by significant premorbid IQ variations.

The spatial span subtest of the Wechsler Memory Scale—Revised (WMS) (32) was used to assess visuospatial working memory. Working memory is one of the most commonly described domains of neurocognitive impairment in patients with schizophrenia spectrum disorders (33) and was therefore assessed as one neurocognitive marker in this study.

### Statistical Analysis

This analysis was performed in all participants younger than 50 years to avoid potential bias of older age on ER and SID, since older age is known to impact both ER and even more so SID (2, 18). For this purpose, data of seven healthy controls and seven patients aged 50 years or older were excluded from the analysis. Thus, the study population consists of 31 male controls, 48 female controls, 30 male patients, and 21 female patients. However, note that values of 10 controls for the variable UPSIT are missing, as well as values of 7 controls and 2 cases for the variable FEEL.

The primary question was the influence of “group” (i.e., healthy control vs. patient) and sex on UPSIT and FEEL scores. A MANOVA with UPSIT and FEEL scores as dependent variables was calculated considering the main effects “group” and “sex,” as well as the interaction between group and sex. In case of a non-significant interaction, the factor was removed from the final model. In a second model, age and WMS total score were considered additionally. All analyses were repeated for the single target variables UPSIT and FEEL [analyses of variance (ANOVAs)]. ANOVAS were calculated as exploratory analyses for the target variables WMS, MWT, and age with main effects “group” and “sex” as well as their interaction. Pearson correlations were calculated between FEEL, UPSIT, PANSS, and WMS scores as well as duration of illness (DUI). Primary and exploratory analyses were performed using the R program, version 3.3.1^®^. Further descriptive analyses, correlations, Chi-square tests, and *t*-tests were performed using IBM SPSS Statistics Version 24^®^. Effect sizes were calculated using freely available online effect size calculators. All tests were two sided, and level of significance was *p* < 0.05.

## Results

The sample for this analysis consists of 51 patients with a schizophrenia spectrum disorder (41.2% female) and 79 healthy controls (60.8% female). There was a significant difference in age between patients and controls (*p* = 0.002) but not between women and men (see Tables 1 and 2).

**Table 1:** Sociodemographic data of patients and controls.

	Sex	Group		Significant*p* values shown
Civil status	Male	Controls	90.3% never married, 6.5% married, 3.2% partnership	
		Patients	90% never married, 10% married	

	Female	Controls	85.4% never married, 8.3% married, 2.1% partnership, 4.2% divorced	
		Patients	66.7% never married, 28.6% married, 4.8% divorced	

	Total	Controls	87.3% never married, 7.6% married, 2.5% divorced, 2.5% partnership	
		Patients	80.4% never married, 17.6% married, 2% divorced	

Highest completed education	Male	Controls	64.5% 12 years and diploma, 12.9% apprenticeship, 22.6% college/university	Male HC vs. Pat.: 0.004
		Patients	36.7% 9 years, 30% 12 years and diploma, 23.3% apprenticeship, 10% college/university	

	Female	Controls	2.1% 9 years, 75% 12 years and diploma, 6.3% apprenticeship, 16.7% college/university	Female HC vs. Pat.: 0.05
		Patients	14.3% 9 years, 47.6% 12 years and diploma, 19% apprenticeship, 19% college-university	

	Total	Controls	1.3% 9 years, 70.9% 12 years and diploma, 8.9% apprenticeship, 19% college-university	HC vs. Pat.: <0.001
		Patients	27.5% 9 years, 37.3% 12 years and diploma, 21.6% apprenticeship, 13.7% college/university	

Occupation	Male	Controls	29% full-time employment, 3.2% part-time employment, 3.2% unemployed, 64.5% student	HC vs. Pat.: <0.001
		Patients	10% full-time employment, 36.7% unemployed, 3.3% marginal employment, 20% student, 30% retirement	

	Female	Controls	22.9% full-time employment, 8.3% part-time employment, 68.8% student	Female HC vs. Pat.: <0.001
		Patients	19% full-time employment, 9.5% part-time employment, 14.3% unemployed, 19% student, 28.6% retirement, 9.5% unknown	

	Total	Controls	25.3% full-time employment, 6.3% part-time employment, 1.3% unemployed, 67.1% student	HC vs. Pat.: <0.001
		Patients	13.7% full-time employment, 3.9% part-time employment, 2% marginal employment, 27.5% unemployed, 19.6% student, 29.4% retirement	

*HC, healthy control group; Pat., patient group*.

**Table 2:** Age and substance use in patients and controls.

	Sex	Group		Significant *p* values shown
Lifetime substance use (yes)^a^	Male	Controls	38.7%	Male HC vs. Pat.: 0.002
		Patients	83.3%	
	Female	Controls	35.4%	Female HC vs. Pat.: 0.035
		Patients	65%	
	Total	Controls	36.7%	HC vs. Pat.: <0.001
		Patients	76%	

Smoking	Male	Controls	32.3%	Male HC vs. Pat.: 0.002
		Patients	76.7%	
	Female	Controls	33.3%	Female HC vs. Pat.: 0.036
		Patients	61.9%	
	Total	Controls	32.9%	HC vs. Pat.: <0.001
		Patients	70.6%	

Age	Male	Controls	26.65% (SD, 6.04)	Male HC vs. Pat.: 0.038
		Patients	30.67% (SD. 8.59)	
	Female	Controls	27.81% (SD, 8,86)	Female HC vs. Pat.: 0.022
		Patients	33.33% (SD, 9.4)	
	Total	Controls	27.35% (SD, 7.85)	HC vs. Pat.: 0.004
		Patients	31.76% (SD, 8.94)	

*^a^Including any of the following substances: nicotine, alcohol, cannabis, cocaine, amphetamines, MDMA, heroine, psychedelics, or other unspecified substances; current use of non-prescribed drugs or alcohol at the time of assessment was an exclusion criterion*.

*HC, healthy control group; Pat., patient group*.

Group category had a highly significant influence on WMS total scores (*p* < 0.0001), whereas sex narrowly reached significance level (*p* = 0.05). Neither sex nor group had a significant influence on MWT-B scores. See Tables 3 and 4 for detailed scores.

**Table 3:** Mean values, SDs for FEEL, and subscores and UPSIT.

	Sex	Group	Mean	SD
FEEL total score	Male	Controls	34.53	3.7
		Patients	31.85	5.81
	Female	Controls	35.12	4.58
		Patients	31.25	5.38
	Total	Controls	34.94	4.26
		Patients	31.59	5.58

Fear	Male	Controls	4.81	1.69
		Patients	4.54	1.67
	Female	Controls	4.84	1.75
		Patients	3.90	2.01
	Total	Controls	4.83	1.71
		Patients	4.27	1.86

Joy	Male	Controls	6.78	0.42
		Patients	6.68	0.67
	Female	Controls	6.82	0.44
		Patients	6.76	0.44
	Total	Controls	6.81	0.58
		Patients	6.71	0.95

Surprise	Male	Controls	5.89	1.12
		Patients	5.89	1.26
	Female	Controls	6.40	0.78
		Patients	6.33	0.86
	Total	Controls	6.21	0.95
		Patients	6.08	1.12

Disgust	Male	Controls	5.26	1.51
		Patients	4.32	1.93
	Female	Controls	5.40	1.72
		Patients	4.52	2.09
	Total	Controls	5.35	1.64
		Patients	4.41	1.98

Sadness	Male	Controls	6	1.44
		Patients	5.32	1.91
	Female	Controls	5.84	1.45
		Patients	4.62	1.66
	Total	Controls	5.90	1.44
		Patients	5.02	1.82

Anger	Male	Controls	6.37	0.89
		Patients	5.68	1.66
	Female	Controls	6.42	0.89
		Patients	5.90	1.18
	Total	Controls	6.40	0.88
		Patients	5.78	1.46

UPSIT	Male	Controls	33.44	3.38
		Patients	32.97	3.64
	Female	Controls	35.64	2.66
		Patients	32.76	4.23
	Total	Controls	34.78	3.13
		Patients	32.88	3.86

**Table 4:** Means and SDs for working memory (WMS) and IQ task (MWT-B).

WMS forward	Male	Controls	11.39	1.36
		Patients	9.83	2.21
	Female	Controls	10.81	1.68
		Patients	9	2.53
	Total	Controls	11.04	1.57
		Patients	9.48	2.35

WMS backward	Male	Controls	9.94	1.5
		Patients	7.57	2.79
	Female	Controls	9.26	1.86
		Patients	7	2.42
	Total	Controls	9.53	1.75
		Patients	7.33	2.62

WMS total score	Male	Controls	21.29	2.48
		Patients	17.39	4.77
	Female	Controls	20.06	2.98
		Patients	16	4.48
	Total	Controls	20.55	2.84
		Patients	16.8	4.64

MWT-B	Male	Controls	29.03	4.47
		Patients	29.3	3.89
	Female	Controls	30.25	3.32
		Patients	28.76	4.91
	Total	Controls	29.77	3.83
		Patients	29.10	4.29

### Patients’ Characteristics

Distribution of diagnoses was 43.1% schizophrenia, 23.5% schizophreniform disorder, and 33.3% schizoaffective disorder. There was no significant difference in distribution of diagnoses between female and male patients. Also, there were no significant differences in patients’ characteristics between female and male patients (see Table 5).

**Table 5:** Description of patient group.

PANSS-positive score	Male	14.10 (SD 5.14)
	Female	11.90 (SD 4.15)
PANSS-negative score	Male	18.0.3 (SD 4.34)
	Female	15.8 (SD 7.8)
PANSS global score	Male	32.43 (SD 6.8)
	Female	30.30 (SD 10.19)
PANSS total score	Male	64.57 (SD 11.89)
	Female	58 (SD 18.91)
History of inpatient admission (yes)	Male	96.7%
	Female	100%
Number of inpatient admissions	Male	4.33 (SD 8)
	Female	3.48 (SD 3.46)
Antipsychotic medication (yes)	Male	96.7%
	Female	95%
More than 1 AP prescribed (low-potency excluded)	Male	42.9%
	Female	47.4%
Type of AP (low-potency excluded)	Male	SGA only 62.1%
		SGA and FGA combined 24.1%
		FGA only 13.8%
	Female	SGA only 63.2%
		SGA and FGA combined 31.6%
		FGA only 5.3%
Clozapine	Male	13.8%
	Female	5.3%
Mood stabilizers	Male	13.3%
	Female	20%
Anxiolytics	Male	33.3%
	Female	40%
Anticholinergic agents	Male	20%
	Female	20%
Antidepressants	Male	40%
	Female	60%
Suicide attempts	Male	33.3%
	Female	57.1%
Psychiatric family history	Male	73.3%
	Female	75%
Duration of illness (months)	Male	72.18 (SD 81.1)
	Female	69.64 (SD 60.5)

*PANSS, Positive and Negative Syndrome Scale; AP, antipsychotic; FGA, first-generation antipsychotic; SGA, second-generation antipsychotic*.

### Primary Analyses

Univariate analyses for UPSIT and FEEL were performed to analyze the influence of sex and group before multivariate analyses.

#### Smell Identification (UPSIT)

The analysis of variance showed no significant influence of the interaction between sex and age (*p* = 0.062). The ANOVA with the factors sex and group on UPSIT showed that controls have significantly higher UPSIT scores than patients (*p* = 0.01) with a moderate effect size (Cohen’s *d* = 0.529), and sex was only borderline significant (*p* = 0.07; adjusted *R*^2^, 0.08; *F*-statistic, 6.2 on 2 and 117 df).

In addition, introducing age and WMS scores increased the influence of sex (*p* = 0.01); on the other hand, the previously significant influence of group category vanished due to its strong relationship with WMS, which had a significant impact on UPSIT scores (*p* = 0.03; multiple *R*^2^, 0.2; adjusted *R*^2^, 0.17; *F*-statistic, 6.5 on 4 and 103 DF). Kendall Tau’s correlation coefficient between WMS total score and group was −0.34, with controls having higher scores than patients. WMS was also correlated with age (Pearson’s correlation coefficient, −0.45). If WMS total score were removed from the ANCOVA, group and age would show a significant influence.

The effect size for the difference between UPSIT scores in males was negligible (Cohen’s *d* = 0.1). However, in female controls vs. patients, the effect size was large with a Cohen’s *d* of 0.815. Similarly, differences in UPSIT scores between female controls and male controls had large effect sizes of 0.723, whereas it was negligible with respect to the comparison of sexes in the patient group (Cohen’s *d* = 0.02).

#### Emotion Recognition (FEEL)

The interaction between sex and group had no significant influence on FEEL scores and was thus not considered in the analysis of variance. The results showed that sex had no significant influence on FEEL scores (*p* = 0.89), but group did (*p* = 0.0004), with healthy controls having significantly higher values on the FEEL test than patients with a moderate effect size (Cohen’s *d* = 0.675; adjusted *R*^2^: 0.09; *F*-statistic, 6.9 on 2 and 118 df). Additional consideration of age and WMS resulted in a significant influence of WMS (*p* = 0.01) only.

In addition, the effect size was moderate for the differences in FEEL scores between male controls and patients (Cohen’s *d* = 0.55) and large for that between female controls and patients (Cohen’s *d* = 0.775).

#### Multivariate Analyses of Variance of Sex and Group on UPSIT and FEEL

The interaction of sex and group was not significant neither in the MANOVA nor the MANCOVA, and thus, only main effects were considered. In the MANOVA, only group showed a significant influence on UPSIT and FEEL (*p* = 0.001), which goes along with the univariate results. However, when age and WMS total scores were included as main factors, sex and WMS became significant (*p* = 0.03 and *p* = 0.01, respectively), whereas the influence of group categorization vanished (*p* = 0.24). The influence of age was not significant (*p* = 0.3).

### Further Analyses

#### FEEL Subscores

With respect to FEEL subscores, there were between-group differences between patients and healthy controls with moderate effect sizes for anger (*p* = 0.004, Cohen’s *d* = 0.51), for sadness (*p* = 0.004, Cohen’s *d* = 0.54), and for disgust (*p* = 0.005, Cohen’s *d* = 0.52) only, with patients showing lower scores. Male patients showed lower scores than their healthy counterparts (*p* = 0.05) with a moderate effect size (Cohen’s *d* = 0.54) in recognizing disgust and bordering significance in recognizing anger (*p* = 0.059, Cohen’s *d* = 0.52). The latter was mirrored in female patients with borderline significance for anger (*p* = 0.052, Cohen’s *d* = 0.49) and for fear (*p* = 0.059, Cohen’s *d* = 0.49). However, female patients also had lower scores than their healthy counterparts in recognizing sadness (*p* = 0.003) with a large effect size (Cohen’s *d* = 0.78). There were no significant differences in FEEL subscores between male and female patients. In healthy controls, women had better results in identifying surprise than men (*p* = 0.026, Cohen’s *d* = 0.53).

Both patients and controls reached best performances with respect to identifying happy faces and worse for fearful ones. Males and females in both groups mirrored the results on the hierarchy of positive and negative valences; however, male patients had lowest scores for disgust (see Table 3).

#### Clinical Modulators

Correlation coefficients were calculated between PANSS, estimate of DUI (defined here as the time since appearance of first psychotic symptoms), FEEL, and UPSIT scores to investigate possible modulators.

FEEL and UPSIT correlated significantly with each other (*p* < 0.001, *r* = 0.502). Neither UPSIT nor FEEL correlated significantly with PANSS total scores.

Looking at female and male patients separately, the following results were yielded: both within the male and the female patient group FEEL and UPSIT correlated significantly with each other (males: *r* = 0.487, *p* = 0.009; females: *r* = 0.528, *p* = 0.014), mirroring the results in the entire patient group. However, only in male patients, DUI correlated negatively with both FEEL (*r* = −0.409, *p* = 0.031) and UPSIT (*r* = −0.528, *p* = 0.003). However, only in female patients, there was a correlation between FEEL scores and psychopathology, i.e., PANSS total (*r* = −0.526, *p* = 0.017) and PANSS-negative score (*r* = −0.696, *p* = 0.001).

However, both UPSIT and FEEL correlated positively with WMS total scores (WMS-UPSIT *p* = 0.001, *r* = 0.328; WMS-FEEL *p* = 0.000, *r* = 0.411). Specifically, WMS total score correlated positively with FEEL in the female patient group (*p* = 0.010, *r* = 0.608) and with UPSIT in the male patient group (*p* = 0.003, *r* = 0.585). Correlations between WMS total score and UPSIT or FEEL were non-significant in all other subgroups.

## Discussion

The first finding of this analysis is that being affected by a schizophrenia spectrum disorders impact one’s ability to correctly recognize facial affects and identify odors. Indeed, patients performed worse than healthy controls in both tasks. Deficits in ER and SID in patients with schizophrenia are quite a robust finding (2, 7), and our results serve to corroborate the validity of our sample with respect to these differences.

However, the impact of sex seems more complex both in the existing literature as well as in our sample. With respect to facial affect recognition, the right-hemispheric hypothesis postulates that men might be more affected by deficits in ER due to a greater activation of right-hemispheric regions during such tasks and greater right-hemispheric dysfunctions in schizophrenia (34). The currently available literature is somewhat inconsistent regarding the direction of sex differences of ER in patients with schizophrenia with some studies reporting a female advantage (10, 35), others, e.g., meta-analytical data revealing no effect of sex (2, 36). As for SI, several previous studies performed in healthy samples and in patients with schizophrenia have described subtly better olfactory identification performances in women (8). In our analysis, effects of sex were only borderline significant for UPSIT and not significant for FEEL scores in the univariate analyses, with healthy women having slightly higher scores in both tasks with large effect sizes compared to their diseased counterparts. In addition, healthy women also recognized odors better than their male counterparts, suggesting that the possible female superiority in these tasks might not be only illness related. As a matter of the fact, the multivariate analysis revealed that sex-related effects might become more relevant when additional, potentially influencing factors, such as age and visuospatial working memory, are accounted for. Our results suggest that sex and working memory capacities together might possibly supersede direct effects related to the illness itself. Indeed, both SID and ER scores correlated with visuospatial working memory results. Mind you, however, that working memory itself is strongly affected by illness, with working memory capacities being worse in patients than in controls for both sexes; this impairment being a robust finding in schizophrenia (13). Hence, these findings suggest that sex-related differences in ER and SI tasks in patients with schizophrenia spectrum disorders might need to be interpreted within the context of working memory capacities. ER is acknowledged as a social cognitive ability in the larger sense of the term and SID can be seen as a higher cognitive function, which similarly to ER requires neurocognitive and social cognitive skills. In this context, our results can be cautiously interpreted as evidence for these tasks being indeed complex social cognitions related to neurocognitive functions and being subject to more complex sex-related effects. Also, this analysis reveals that ER and SID are intertwined, i.e., correlated with each other in both sexes, suggesting again that they might underlie similar pathomechanisms in the context of schizophrenia spectrum disorders.

Interestingly, in male patients only, both SID and ER tasks were related to DUI, i.e., the longer the DUI, the stronger the impairment in both tasks, hinting to an effect of chronicity of illness on these abilities that women might be able to compensate somewhat better. However, in female patients, current psychopathology (represented by the PANSS total score) and negative symptoms (PANSS negative score) correlated negatively with facial affect recognition, underlining the notion of the effect of immediate psychopathology on this social cognitive task. Thus, our results might suggest a more complex relationship with sex, which goes beyond a mere dichotomy of superiority or inferiority.

With respect to emotional valence, both groups performed best at recognizing happy faces and identifying fear seemed most difficult to most. Specifically, patients had more difficulties recognizing the negative emotions anger, sadness, and disgust than controls with a differential pattern for men (with disgust and anger being the emotions with the largest difference to healthy controls) and women (with anger, fear, and sadness, respectively).

The more accurate identification of emotionally positive faces and higher number of errors in recognizing negative emotions is a pattern that has been shown in healthy populations and in patients with schizophrenia spectrum disorders (37, 38). The literature supports indeed the notion that negative emotions are more difficult to recognize than positive ones (39, 40). In patients, abnormalities in self-representation and causal attributions that might include misinterpreting negative emotions of others more easily have been postulated to facilitate the development of paranoid ideas alongside a certain degree of distortion of the perception of reality (41). Difficulties correctly identifying facial affects of others might easily lead one to misinterpret facial cues and overestimate or underestimate reactions of social friendliness or adversity, resulting in either suspiciousness or an increased vulnerability to being victimized.

Several limitations that may influence the interpretation of our findings must be addressed. First, the sample size, especially the female sample, is rather small, and some effects might have been concealed. However, most of those differences that were significant had moderate to large effect sizes. Second, patients and controls differed in age; however, the multivariate analysis excluded remaining relevant effects of age. Also, other features of the olfactory system, such as odor threshold sensitivity, unirhinal presentation of odors, which might have allowed for a more differentiated, hemispheric-sensitive appraisal of potential SIDs, and separate analysis of odor valence were not assessed. Menstrual cycle was not assessed; however, most authors perceive a reduction of olfactory functions to mere effects of reproductive hormones as an oversimplification (8). Although we did not specifically correct for type of antipsychotic, there was no correlation between type of antipsychotic (i.e., atypical vs. typical) or monopharmacy vs. polypharmacy and ER or SID test scores. This is somewhat in contrast with meta-analytical data in ER, where greater impairments have been shown with first-generation antipsychotics (2). Of note, there were no significant differences between male and female patients in patients’ characteristics. Also, there were no associations between diagnosis and SID or ER task results for either sex. Testing situation and sex of participant vs. rater was not assessed separately. However, it can be safely assumed that testing situations were fairly similar for all participants, since all active raters were women, and all assessments were conducted in a quiet room without other observers. Nevertheless, it cannot be excluded that a gender-related bias came into play. As a matter of fact, men have been shown to increase their efforts to impress women in competitive situations with a female observer; however, these efforts might rather tax actual cognitive capacities (42). Finally, other groups have suggested that sex of poser vs. that of observer, which we did not assess, might play a role in ER (43). Thus, the results of our analysis must be interpreted in light of these limitations.

In conclusion, both social cognitive abilities assessed here, ER, and SI were shown to being associated with each other in male and in female patients with schizophrenia spectrum disorders with a strong contribution of effects of working memory. Indeed, effects of sex were only evident when taking into account working memory capacities. Differential effects of modulators on these tasks were seen in women vs. men. Converging evidence of this analysis and of previous studies suggests a link between basic neurocognitive and social cognitive functions in patients with schizophrenia spectrum disorders. Further research to evaluate the direction of causality and the specificity of these findings, as well as the magnitude of the effect of modulating factors, should be conducted. Also, a stronger focus on investigating sex vs. gender effects might help clarify some of the reported inconsistencies.

## Ethics Statement

This study was carried out in accordance with the recommendations of the Ethics Committee of the Medical University of Vienna, Austria with written informed consent from all subjects. All subjects gave written informed consent in accordance with the Declaration of Helsinki. The protocol was approved by the Ethics Committee of the Medical University of Vienna, Austria.

## Author Contributions

NM designed, implemented, and managed the study as Co-PI; was actively involved in participants’ recruitment, interrater training, data management, statistical analysis, and interpretation; and; drafted and elaborated all versions of the manuscript. RK and MS designed and implemented the study, were actively involved in recruitment, data acquisition and analysis, interpretation, and revision of the manuscript. TA, CH, and AG were actively involved in data acquisition, recruitment, assessments, interpretation of data, and revising the manuscript. SZ was involved in the concept and design of the original study, data analysis, statistical calculations, interpretation of data, and critical revisions of the manuscript. HH and HT were involved in the design of the study, provision of assessment instruments, data interpretation and revision of manuscript. HA designed, managed, and supervised the study as Co-PI; contributed substantially to data interpretation; and critically revised all version of the manuscript. All authors contributed to and approved of the final version of the manuscript.

## Conflict of Interest Statement

The authors declare that the research was conducted in the absence of any commercial or financial relationships that could be construed as a potential conflict of interest.
